# Research trends and hotspots of adult Alagille syndrome: a bibliometric analysis

**DOI:** 10.1186/s13023-025-03855-5

**Published:** 2025-07-01

**Authors:** Qing Liu, Jie Yang, Wentian Liu, Chao Sun

**Affiliations:** 1 Department of Gastroenterology, Tianjin Medical University General Hospital Airport Hospital, East Street 6, Tianjin Airport Economic Area, Tianjin, 300308 China; 2 Department of Gastroenterology and Hepatology, Tianjin Medical University General Hospital, Anshan Road 154, Heping District, Tianjin, 300052 China

**Keywords:** Alagille syndrome, Rare disorder, Bibliometric, Cite space, VOS viewer

## Abstract

**Introduction:**

Alagille syndrome (ALGS) is a complex and rare autosomal dominant disorder that affects multiple systemic organs, including the liver, heart, kidneys, blood vessels, and eyes. However, as a rare disease, adult ALGS is still underrecognized clinically. The aim of this article is to review the research status and trends of adult ALGS worldwide in the existing literature.

**Methods:**

The authors utilized the Web of Science Core Collection database to identify articles on adult ALGS published from the database inception to December 31, 2023. The authors used bibliometric methods to analyze authors, institutions, countries, journals, and references of 156 included articles.

**Results:**

The number of publications in this field has shown a volatile growth trend. The United States published the most articles and University of Pennsylvania is the leading institution in this area. The research on adult ALGS mainly focuses on molecular biology, genetics, health, nursing and clinical medicine. The keywords were “human jagged1,” mutations” and “children”. The keyword with the strongest citation burst is “children”.

**Conclusions:**

Our study systematically summarizes the results of adult ALGS researches, describes and predicts research hotspots and trends on a global scale, which may be helpful for clinicians and researchers to improve their clinical understanding of this disease, and provide a valuable reference for future intensive investigation.

## Introduction

Alagille syndrome (ALGS) is a complex and rare autosomal dominant genetic disease that involves multiple system organs, including liver, heart, kidneys, blood vessels, eyes, etc. Alagille syndrome was first reported in 1969 by Alagille et al. [1] and was proposed again in 1975 alongside diagnostic criteria establishment [2]. ALGS is generally considered a childhood disease, and the majority of research has been carried out among pediatric patients. Molecular studies have addressed that the disease is attributed to active disease-causing gene mutations in the intercellular *NOTCH* signaling pathway-*JAGGED1 (JAG1)* and *NOTCH2*, most commonly *JAG1* mutations (ALGS type 1), but in a fraction of subjects manifested as *NOTCH2* mutations (ALGS type 2). Those mutated genes are inherited in an autosomal dominant manner with incomplete penetrance, which leads to a highly variable disease phenotype, organ involvement as well as disease severity [3–5]. ALGS is often misdiagnosed in adults due to lacking of awareness and heterogeneous clinical manifestations. However, some studies have shown that about 90% of children with ALGS survive after 18 years of age irrespective of cholestasis [6]. Therefore, in fact, adult patients with ALGS are not uncommon pertinent to incidence, and ALGS should not be conceptualized as only a childhood disease. Taken together, it is of great significance regarding the early diagnosis, effective treatment and prompt intervention against ALGS in adults.

Bibliometrics is a methodology for analyzing the extant literature. It examines the output and status of publications within a specific research field from both quantitative and qualitative perspectives. Over time, bibliometric analysis has emerged as one of the most widely adopted approaches to evaluate the credibility, quality, and impact of academic works [7, 8]. Although bibliometrics is not an infallible technique, it serves as a valuable tool for guiding funding agencies to allocate resources and identify areas within disciplines that may be under-researched. It enables researchers and scholars to gain deeper insights into the academic landscape, recognize emerging trends, and assess the influence of different works within their respective fields [9].

At present, there is lacking of bibliometric analysis pertaining to adult ALGS. This paper makes a preliminary evaluation, highlights prospect of the research status, addresses recent development and future perspective of adult Alagille syndrome by using bibliometric methods, and visualizes it with the purpose of providing information and directing future clinical and scientific research in the context of ALGS.

## Materials and methods

### Database and search strategy

We enter the subject terms into the Web of Science (WOS) Core Collection TS = (adults) OR TS = (adolescence) OR TS = (“grow up”)) AND (TS = (Alagille) OR TS = (“Alagille Syndrome”) OR TS = (ALGS). The search period is from inception of the Web of Science database to December 31, 2023. The literature types were restrained to article and review, and the inclusion criteria were: related studies investigating the pathogenesis, etiology, incidence, prevention, diagnosis, treatment and prognosis in the context of adult ALGS. The study design type was not limited. Exclusion criteria: ① ALGS in children alone and literature irrelevant to adult ALGS; ② Repeatedly published literature, conference papers, dissertations, news and newspapers, etc.

### Data analysis

The title, author, year of publication, country, institution, keywords, citations, abstracts, and references are obtained from the WOS database. The downloaded file is in plain text format. Microsoft Excel 2019 was used to count the publications’ numbers, literature types and journal sources of adult ALGS. VOSviewer1.6.20, Charticulator and ArcMap10.8 were used to visualize the national and regional distribution, cooperation and key words of adult ALGS-related researches. Cluster analysis of co-cited literature was performed using Cite Space 6.3.R3.

## Results

### Analysis of the numbers of publication

**Fig. 1:**
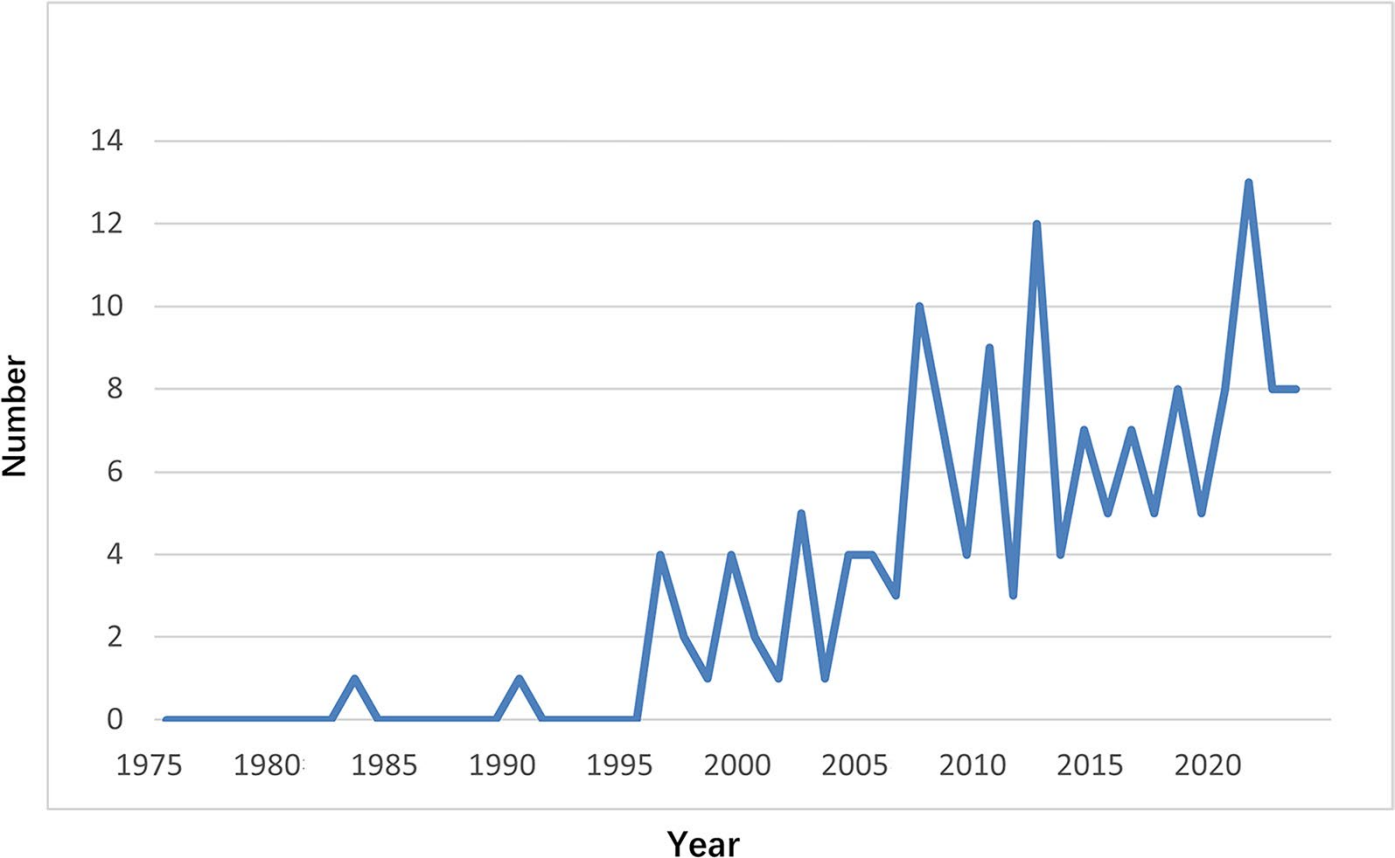
Distribution annual numbers of publication of research literature on adult Alagille syndrome from 1975 to 2023

One hundred sixty-five articles were retrieved from WOS database, excluding repeated articles, conference papers, dissertations, news and newspapers. Finally, 156 articles were involved, consisting of 52 reviews (33.3%) and 104 articles (66.7%) in addition to 15 case reports (9.6%). The numbers of literature regarding adult ALGS have been on the rise since 1975 when looking into the general trend, which can be roughly divided into three development stages with distinct characteristics as follows: ① From 1975 to 1995: Since 1975, ALGS was put forward again and diagnostic criteria was established. ALGS was usually considered as a childhood disease, thus the most studies focused on children. Reports on adult ALGS were very rare. ② From 1996 to 2006, the numbers of literature in relation to adult ALGS increased slightly compared with the previous period, with the numbers of publication fluctuating between 1 and 4 per year. ③ From 2007 to 2023, the overall literature on adult ALGS showed a volatile increase compared with before, with the maximum number of publications per year being 13 (Fig. 1).

### Analysis of the literature co-citation

**Fig. 2:**
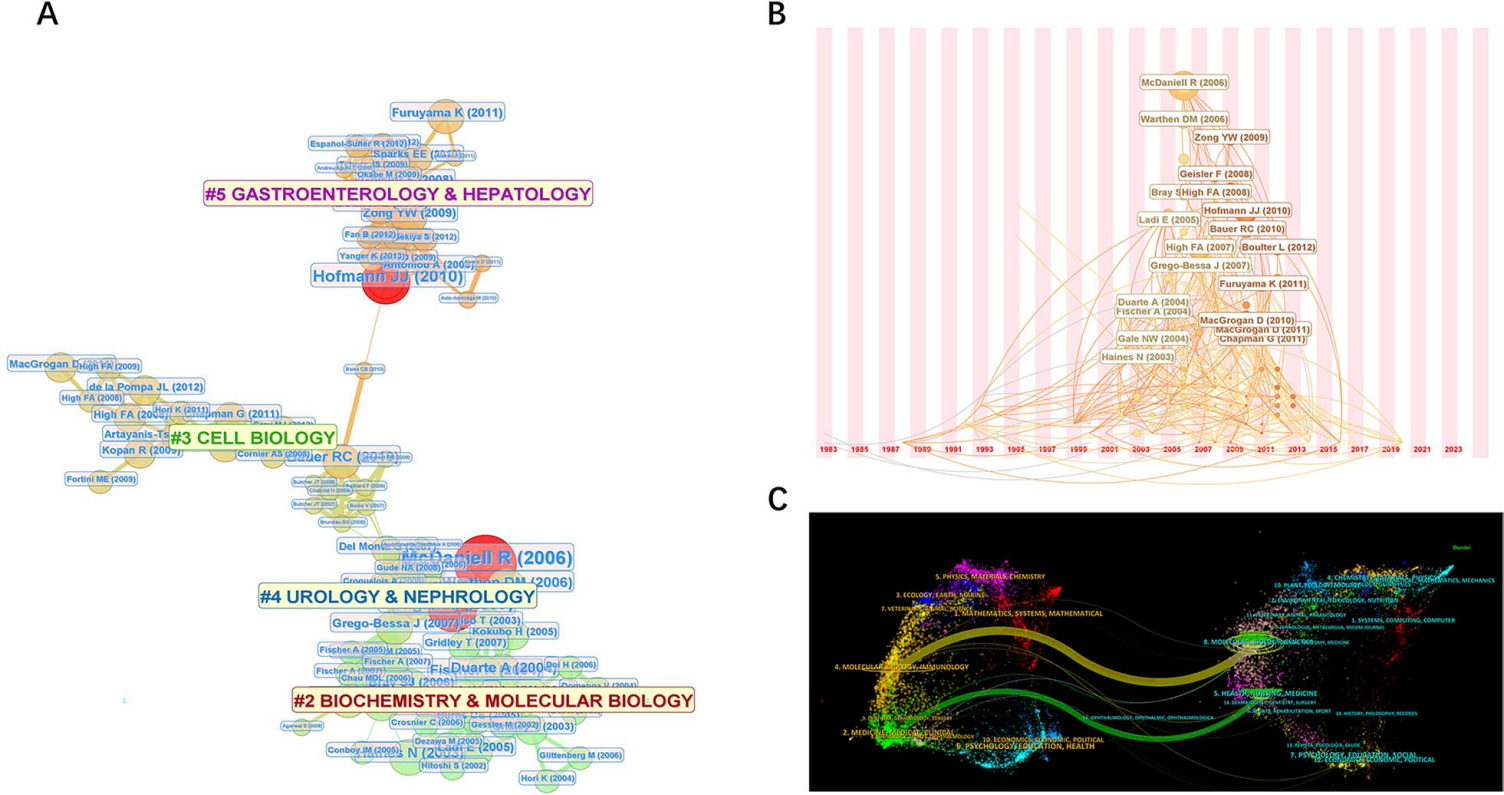
Visualization map of literature co-citation clustering (**A**) and timeline (**B**). The dual-map overlay of journals related to adult Alagille syndrome (**C**)

We analyzed 367 co-cited literatures, performed cluster analysis through Cite Space, and adopted LLR algorithm. N is the total number of nodes and E is the total number of connections in the network, indicative of the co-cited relationship; Network density indicates how tightly connected nodes are. A total of nodes N = 367, connection E = 889, network density 0.0132, four effective clusters were obtained by clustering, and the module value Q = 0.9104 and the contour value S = 0.9574 of the clusters were obtained, which showed that the clustering structure was significant and convincing. The research content of the co-cited literature covers clusters # 2 BIOCHEMISTEY & MOLECULAR BIOLOGY, # 3 CELL BIOLOGY, # 4 UROLOGY & NEPHROLOGY, and # 5 GASTROENTEROLOGY & HEPATOLOGY. The smaller serial number of cluster represent field with more co-citations included. Journals in field of BIOCHEMISTEY & MOLECULAR BIOLOGY have the most co-citations. Among them, McDaniell [5] published a very important paper in AMERICAN JOURNAL OF HUMAN GENETICS with important influence in this field (Fig. 2A). It can be seen that the number of citations of literature published from 2000 to 2014 is significantly higher than that of previously published literatures, which may indicate that the quality of articles published during this period is higher than before (Fig. 2B).

### Analysis of the source distribution

In order to facilitate researchers to inquire about relevant research results and obtain the latest research progress in this field, we analyzed the journals included in the literature sources. The most published journal is HEPATOLOGY, which is a Zone 1 professional journal in the field of liver diseases. It mainly publishes original and peer-reviewed articles on various aspects of liver structure, function and diseases. Since its founding in 1981, it has been indexed in well-known retrieval systems in domestic and overseas such as SCIE (Science Citation Index Extension Board). The second place is JOURNAL OF PEDIATRIC GASTROENTEROLOGY AND NUTRITION, followed by CIRCULATION RESEARCH and GASTROENTEROLOGY with 5 articles, respectively. The total numbers of citation covering these 159 literatures in the field is 10,623, the average number of citations is 68.1, and the h-index is 52 (Table 1).

**Table 1:** The top 5 journals related to adult Alagille syndrome research from 1975 to 2023

Journal Name	Number of articles published	Percentage (%)	Impact factor (2024)
HEPATOLOGY	10	6.41	12.9
JOURNAL OF PEDIATRIC GASTROENTEROLOGY AND NUTRITION	6	3.85	2.4
CIRCULATION RESEARCH	5	3.21	16.5
GASTROENTEROLOGY	5	3.21	25.7
CURRENT TOPICS IN DEVELOPMENTAL BIOLOGY	4	2.56	5.242

We employed a dual-map overlay atlas through Cite Space. In Fig. 2C, the citing journal was represented by the geographic area on the left side, and the cited journal was represented by the map on the right. The connection line served as the relationship between the citing journal and the cited journal. The thicker shades of connection line represent literature with closer relationship between the two fields. The research on adult ALGS mainly focuses on molecular biology, genetics, health, nursing and clinical medicine.

### Analysis of author and institution

**Fig. 3:**
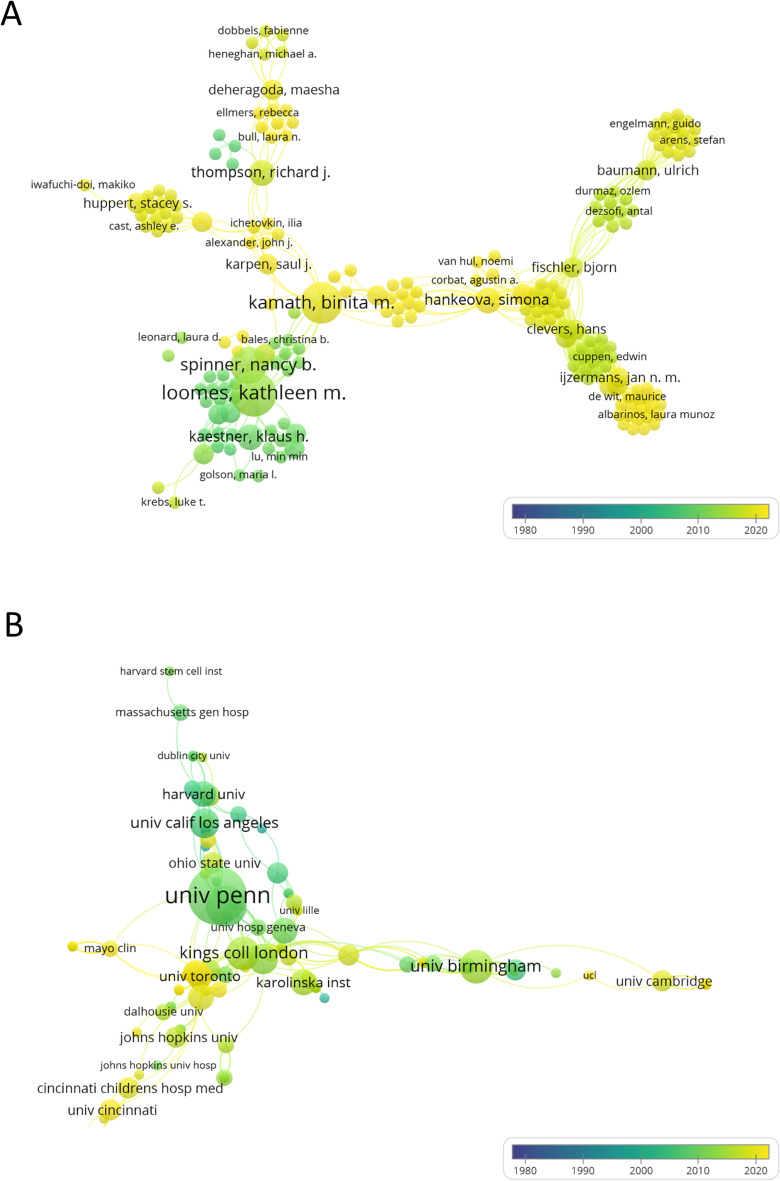
Network visualization map of the authors (**A**) and institutions (**B**) collaboration

The visualization map of the cooperation network published by the authors and the institutions was shown in Fig. 3. The authors who have published ≥ 3 articles were shown in Table 2, referring to Kathleen M Loomes, Binita M. Kamath, and Nancy B. Spinner with the specific articles number published as 7, 6 and 5, respectively. Among them, Kathleen M Loomes was the author who published the most articles and co-published articles with other authors. The involved literatures were from a total of 281 institutions. The institutions that have published 5 or more articles were the University of Pennsylvania, Children’s Hospital of Philadelphia, Kings College London, the University of Birmingham, Kings College London Hospital and the University of California, Los Angeles (Table 3). Among them, the University of Pennsylvania had the largest numbers of article published, the numbers of citation and cooperation with other institutions also ranked first.

**Table 2:** Authors with ≥ 3 published articles

Author	Number of articles published	Citation (times)	Total link strength
Kathleen M Loomes	7	394	50
Binita M. Kamath	6	132	40
Nancy B. Spinner	5	404	31
Donal MacGrogan	4	458	9
Simona Hankeova	3	88	39
Freddy Radtke	3	241	23
Richard J Thompson	3	53	21
Klaus H Kaestner	3	253	20
Luke Boulter	3	658	17
José Luis de la Pompa	3	400	7

**Table 3:** Institutions with ≥ 5 published articles

Name of Institution	Number of articles published	Citation (times)	Total link strength
University of Pennsylvania	14	1523	37
Philadelphia Children’s	8	978	24
Kings College London	6	281	31
University of Birmingham	6	239	19
Kings College Hospital London	5	190	18
University of California, Los Angeles	5	582	5

### Analysis of the geographical of countries

**Fig. 4:**
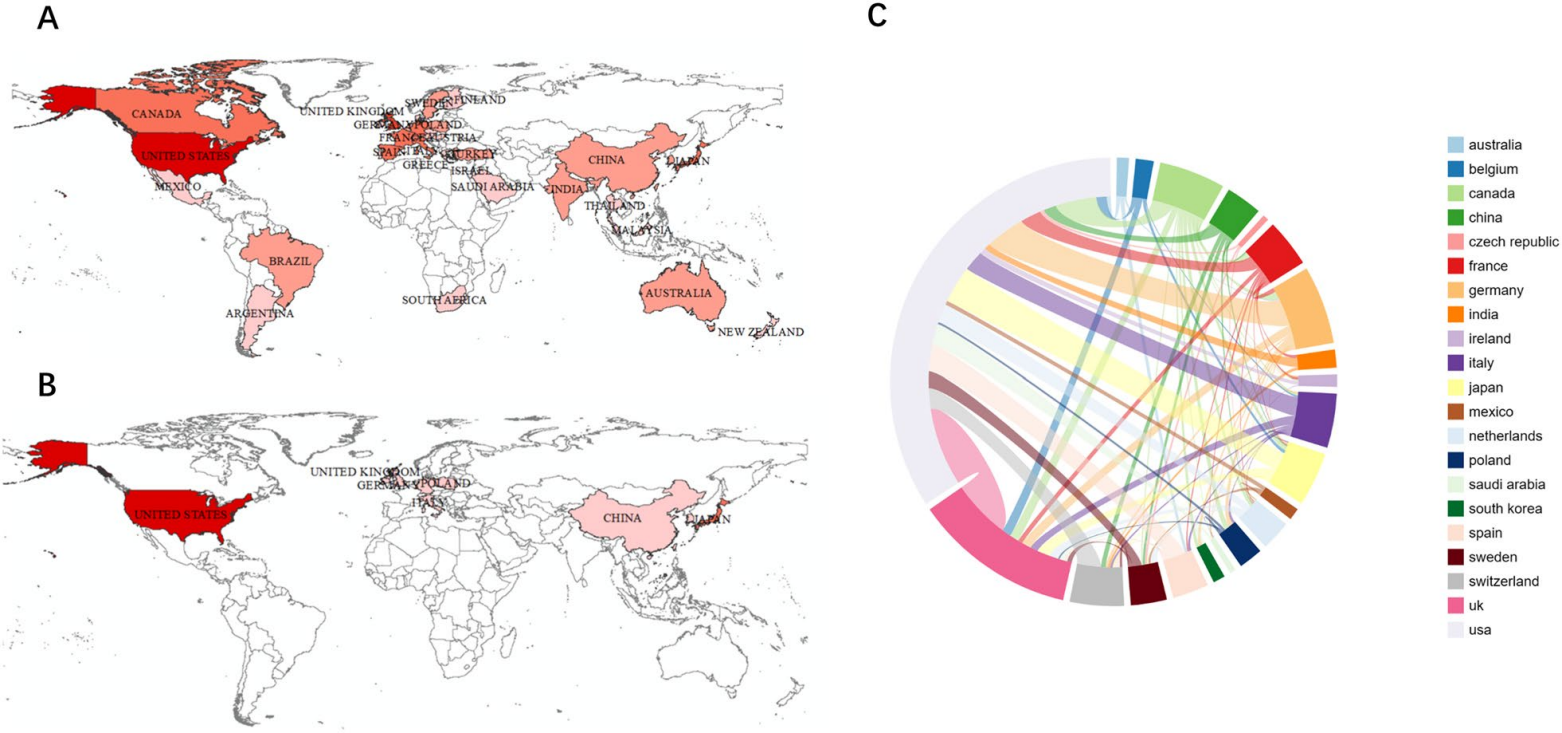
Geographic distribution of global articles (**A**) and cases (**B**) on adult Alagille syndrome. Cooperation status of countries published research literature on adult Alagille syndrome (**C**)

Figure 4 shows the participation of 21 countries and regions we have analyzed in the geographical distribution of global publications in the realm of adult ALGS. The United States has published the most studies in this field (79 articles), followed by the United Kingdom (30 articles) and Germany (15 articles) (Fig. 4A). Many countries have also reported scatter cases of adult ALGS. In terms of the numbers of case reported, the United States still ranks first (7 articles), followed by Japan (3 articles) and the United Kingdom (2 articles). China, Germany, Ireland, Italy, Poland and South Korea each have one case report (Fig. 4B).

By drawing the macro-country cooperation network (Fig. 4C) and analyzing the distribution and cooperation of research forces in the field of adult ALGS from 1975 to 2023, it can be seen that the United States, the United Kingdom, and Germany, which ranked among the top three in terms of published articles, were not solely completed by themselves. In addition to related research, they were also actively cooperating with other countries and institutions. China has also made some research progress in this field, but there were few related studies and case reports released, which showed that China lacked relevant leading institutions, had little cooperation with other countries, and its collective advantages were not obvious.

### Analysis of keywords

**Fig. 5:**
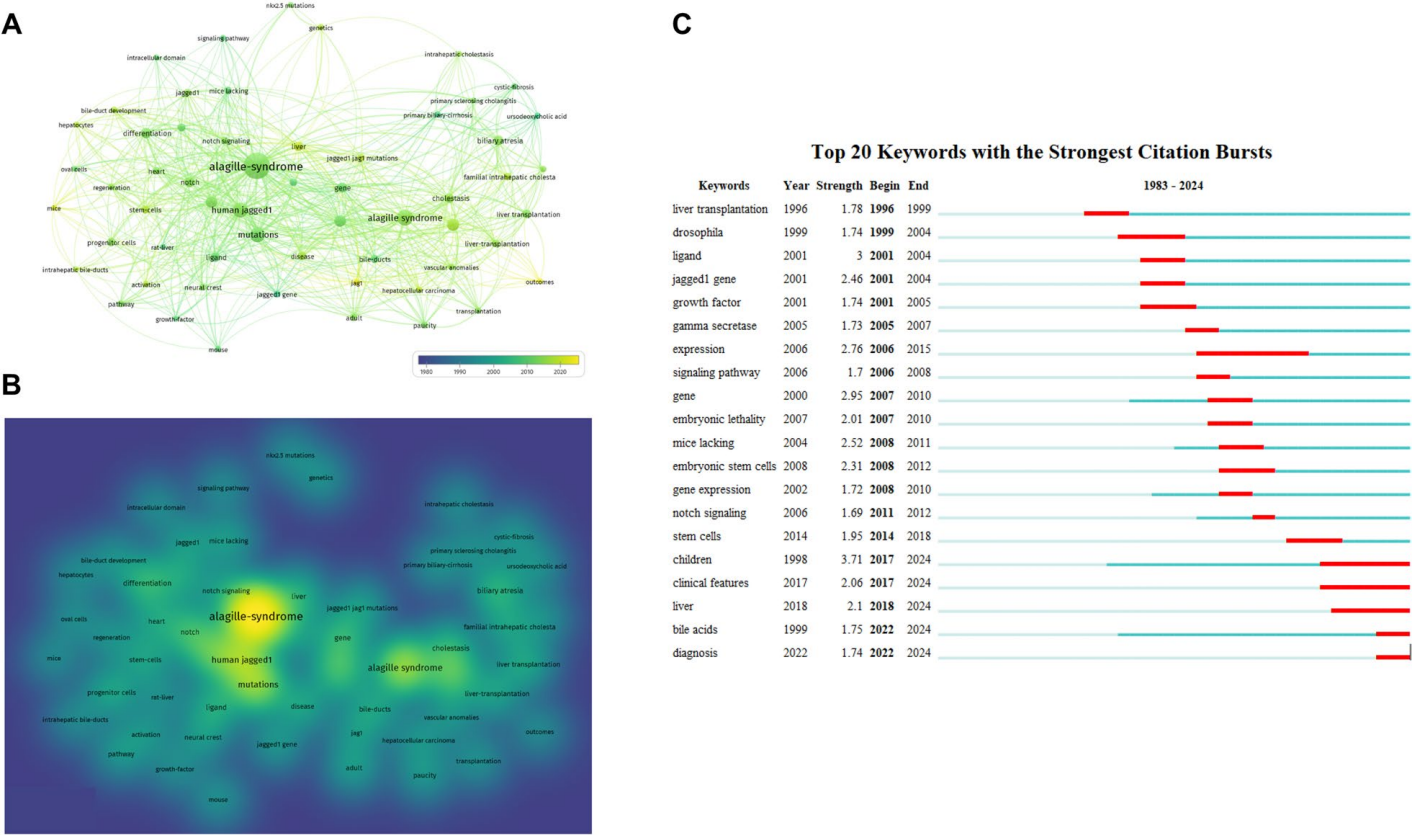
Overlay and density visualization map of the keywords (**A**, **B**), and keywords with the strongest citation bursts (**C**). The color of the nodes indicates the average year of publication, from 1980 to 2013, when the color changed from dark blue to yellow. Keywords appearing in yellow fluorescent areas appear more frequently

Keywords can reflect the topic of an article by summarizing its research focus and essentials. Therefore, keywords with high frequency can represent hotspots of research in this field. In this study, VOS viewer was used to visually analyze the 156 involved articles. Among the 936 keywords in the collinear network, we selected 58 keywords with frequencies more than 5 times. The top 5 keywords according to the total link strength were Alagille-syndrome, human jagged1, mutations, human Alagille syndrome, children (Table 4, Fig. 5). It can be seen that the research hotspot of adult ALGS is the mutation of related genes, and even in the literature related to adult ALGS, the keyword children is still a high-frequency word. Adult ALGS often occurs in childhood and is closely related to childhood ALGS.

**Table 4:** The top 5 total link strength of literature with adult Alagille Syndrome from 1975 to 2023

Keywords	Frequency (times)	Total link strength
Alagille-syndrome	92	289
Human jagged1	29	144
Mutations	30	134
Alagille syndrome	34	133
Children	19	94

## Discussion

ALGS is a complex and rare autosomal dominant genetic disease that involves multiple systems as well as organs, including but not limited to liver, heart, kidneys, blood vessels and eyes. Since ALGS is also a rare disease in China, adult patients with ALGS are often misdiagnosed or overlooked due to a lack of awareness and significant clinical manifestations. Therefore, the diagnosis and treatment of ALGS is still a very important and open topic. This survey uses bibliometric analysis method to analyze the publication year, institution, country, citation and research direction of the existing literature related to adult ALGS in the WOS with the purpose of gaining a preliminary understanding of the current research status and trend in this field.

### General information study

ALGS was first reported by Alagille et al. in 1969 [1] and its preliminary diagnostic criteria was established in 1975 [2]. ALGS is generally considered a rare disease in children, and reports concerning ALGS in adults is scant. It is until 1982 that 13 pediatric hepatologists from the United States and Canada, together with Dr. Alagille and his 8 colleagues from France, reported 15 children and young adults presenting different magnitudes of intrahepatic cholestasis, including two adult patients with ALGS [10]. ALGS is mainly caused by the mutations in two pathogenic genes, that is, *JAG1* and *NOTCH2.* Its clinical manifestations involve multiple systems as well as organs such as the liver, heart, kidney, blood vessels, and eyes. However, the phenotype of ALGS is highly heterogeneous with a lack of typical symptoms in many patients, making it difficult to diagnose [3, 4]. In alignment with the development of molecular biology and the gradual understanding of its pathogenesis by clinicians and researchers, since 1996, the numbers of literature related to adult ALGS have increased slightly, with 1–4 articles published every year. After 2007, the overall literature on adult ALGS showed a volatile increase, with a maximum of 13 articles published annually. According to the growth trend reflected by the curve showing the chronological distribution of literature pertaining to adult ALGS from WOS database, it can be anticipated that the research on adult ALGS will continue to increase, suggesting that adult ALGS has attracted intensive attention as a rare disease.

From the perspective of the distribution of countries conducting research in this field, the top three are the United States, the United Kingdom and Germany. Among these countries, the United States has the most reported cases and documents issued through cooperation with other countries. From the perspective of the distribution of relevant research institutions, compared to other countries, more publications of article with respect to adult ALGS derived from Britain and the United States, suggesting that European and American countries are in leading positions. The highly variable phenotype of ALGS, requiring diagnosis through specific genetic testing, may account for this phenomenon. Considering the rapid development of China’s economy and the progress of molecular diagnostics, ALGS, as a rare disease, has received increasing attention. In this regard, the actual prevalence of this disease is probably much higher than current findings according to epidemiological investigations.

Based on the cited analysis of adult ALGS, most of the highly cited papers are classified into molecular biology, gastroenterology, hepatology, urology and nephrology, implicating that the clinical symptoms of adult ALGS often manifest in the urinary and the liver system. As a result, the research on its pathogenesis, clinical manifestations and molecular diagnosis and treatment is of significant value concerning reference and application.

### Emerging trends, hotspots, and frontiers

Through bibliographic co-citation networks and keyword clustering, a wealth of valuable information in the field of adult ALGS can be systematically collected. This encompasses, but is not limited to, the mechanisms underlying the development of ALGS, the diverse clinical manifestations, disease prognosis, and cutting-edge treatment modalities. In the subsequent sections, we will thoroughly discuss the profound implications of these emerging trends and research hotspots contextually. Additionally, we will explore their potential impacts on future research directions, aiming to provide a comprehensive understanding and guide for further investigations among adult ALGS.

#### Research in molecular diagnostics

Regarding the investigations of ALGS among adults, molecular diagnostics has always been the focus. Both *jagged1* and mutations account for the top position, which shows that genetic testing plays a pivotal role in the diagnosis of this pathophysiological entity. Studies have shown that among individuals with clinical features of ALGS, pathogenic variants of *JAG1* account for the vast majority (94%), while variants of *NOTCH2* are rarely reported (2.5%) [11]. Simultaneous detection of the *JAG1/NOTCH2* genes can identify 97% of the overall subjects [12, 13]. For patients with *JAG1/NOTCH2* gene mutations, ALGS can be diagnosed if they show one or more clinical features concurrently [14]. The heterogeneous clinical presentations of ALGS patients are, to a certain extent, contingent upon whether their condition is triggered by *JAG1* or *NOTCH2* mutations [15]. The incidence rates of liver-related symptoms, including cholestasis and bile duct paucity, are similar in patients with *JAG1-* or *NOTCH2-*associated ALGS. It is worth noting that compared ALGS with *JAG1*, patients with *NOTCH2* variants have a lower penetrance of extrahepatic features, and they seem less likely to present with signs such as facial abnormalities, cardiac abnormalities, posterior embryotoxon, or butterfly vertebrae [6, 11, 14]. The *JAG1-NOTCH* signal transduction directly harnesses the development and homeostasis of the liver and the vascular system, exerting a significant impact on health. Currently, a great deal of research efforts has focused on the signal transduction mechanisms underlying the pathogenesis of ALGS and the genetic modifiers [16, 17]. In the future, it may be possible to regulate the intensity of the Notch signal by utilizing potential therapeutic antibodies, small molecules, and antisense oligonucleotides, thus offering new hope for the treatment of ALGS.

#### Research on the changes of the patient population at different periods

The majority of information pertinent to adult ALGS case stems from subsequent follow-up studies mainly carried out in childhood period [6, 10, 18]. A large international study on the natural course of liver disease in children with ALGS shows that 66.0% of children with ALGS experience ≥ 1 adverse liver-related events before reaching adulthood, such as portal hypertension, liver transplantation, or death. Splenomegaly and thrombocytopenia occur in 65.8% of children with ALGS when they reach adulthood. The level of total serum bilirubin in children with ALGS aged 6 to 12 months has been identified as a predictor of long-term liver prognosis. A median total bilirubin level of < 5.0 mg/dl is significantly associated with a higher natural survival rate, and 79% of these children can survive to adulthood with their own livers [6]. Adult and pediatric patients with ALGS also exhibit distinct clinical manifestations. In children, ALGS typically presents during infancy or early childhood, often prompting medical consultation due to cholestasis. These young patients frequently experience growth retardation and intellectual development delays. Conversely, adult patients usually present with abnormal liver function as the initial symptom, with a relatively lower incidence of cholestasis. Moreover, adult patients often lack typically phenotypic features such as characteristic facial dysmorphology, cardiac anomalies, and ocular abnormalities, which are more commonly observed in pediatric cases. Compared with children, the disease progression in adults places relatively more emphasis on changes in liver function and the development of complications, such as the risk pertaining to cirrhosis and hepatocellular carcinoma (HCC) [19]. HCC is considered a rare complication of ALGS. Unlike pediatric patients, in whom the occurrence of HCC is mostly associated with the background of cirrhosis, the onset and development of HCC in adult patients is mostly irrelevant to cirrhosis [20]. Adult patients with ALGS mostly visit gastroenterology and nephrology departments on account of some atypical symptoms/signs, further verifying the importance and necessity of genetic testing. It should be cautious especially establishing clinical diagnosis of atypical cases, while searching for the evidence of molecular diagnosis is highly recommended.

#### Research on potential treatment regimens

According to the results of our bibliometric analysis, the evidence supporting the treatment of ALGS in adults are limited. Although compare to children, the management of ALGS in adults is not expected to be different, the comorbidities in adults should be taken into account [14]. Treatment of ALGS in adult covers repairing the involved organs such as liver, kidney and heart. Cholestasis represents one of the most important clinical manifestations, therefore the treatment of cholestasis is essential and has received more attention. Supporting nutrition can improve mineral bone disease and metabolic dysregulation concerning the cardiac systems [21], frequently occurring among patients with ALGS. Conventional pharmacological drug against cholestasis in the context of adult ALGS include ursodeoxycholic acid, antihistamines and fibrates. Notably, a novel approved drug for patients with ALGS is Maralixibat (MRX), an ileal bile acid transporter (IBAT) inhibitor, which can reduce the total bile acid pool size through interrupting the enterohepatic circulation of bile acids at the level of ileal enterocytes [22, 23]. Other surgical managements, such as external biliary diversion [24], liver transplantation [25], and the surgeries for relating organs can be applied in patients with serious scenarios [26].

### Clinical implication

This study has clinical implication and practical significance in the following three aspects: (1) Clinicians and junior researchers can effectively acquire basic knowledge of ALGS by studying the research achievements of renowned authors and institutions introduced in this paper, thereby enhancing their scientific research proficiency and clinical skills. (2) Through journal overlay analysis, we can determine relevant research hotspots and suitable journals. This greatly facilitates researchers in seeking potential collaboration opportunities among global scientific research teams with respect to the sharing of rare disease cases and joint efforts to tackle key problems. (3) Through co-citation analysis and keyword co-occurrence analysis, we can more precisely grasp the research trends related to adult ALGS, identify unresolved issues, provide a basis for the rational allocation of scientific research resources, formulate more targeted policies, and accelerate breakthroughs in diagnostic technologies for rare liver diseases and progress in drug research and development.

### Limitation

Our study also has some limitations. First, this paper only analyzes the research in the WOS, but excluding database in Chinese and other language as they have limited functions for bibliometric analysis. This make the results of this study different from the actual situation, but does not affect the knowledge accumulation of the research status worldwide. Second, co-citation analysis may not be the best indicator to measure the value of literature as the new article are less likely to be cited. The co-citation frequency of literature in this research field in the past 10 years is significantly lower than that of previous literature, which does not mean that these literatures are worthless or insignificant in this field. Additionally, there is linguistic bias since the literature selection is restricted to English-language publications only.

Collectively, this literature suggests that molecular diagnosis is indispensible in the context of adult ALGS contemporarily, however, there is knowledge gap regarding its effective treatment. In the future, it is imperative to carry out high-quality, well-designed trials/studies concentrating on breakthroughs of therapeutic strategies against this entity.

## Conclusion

Our study summarizes the results of adult ALGS researches, describes and predicts research hotspots and trends on a global scale, which may be helpful for clinicians and researchers to improve their clinical understanding of this disease, and provide a valuable reference for future intensive investigation.

## Data Availability

Data sharing not applicable to this article as no datasets were generated or analyzed during the current study.

## Abbreviations

ALGS: Alagille syndrome
WOS: Web of Science
HCC: Hepatocellular carcinoma
MRX: Maralixibat
IBAT: Ileal bile acid transporter

## References

[1] Alagille D, Borde J, Habib EC, Thomassin N. Surgical attempts in atresia of the intrahepatic bile ducts with permeable extrahepatic bile duct. Study of 14 cases in children. Arch Fr Pediatr. 1969;26:51–71. 5785517

[2] Alagille D, Odievre M, Gautier M, Dommergues JP. Hepatic ductular hypoplasia associated with characteristic facies, vertebral malformations, retarded physical, mental, and sexual development, and cardiac murmur. J Pediatr. 1975;86:63–71. 803282 10.1016/s0022-3476(75)80706-2

[3] Li L, Krantz ID, Deng Y, et al. Alagille syndrome is caused by mutations in human Jagged1, which encodes a ligand for Notch1. Nat Genet. 1997;16:243–51. 9207788 10.1038/ng0797-243

[4] Oda T, Elkahloun AG, Pike BL, et al. Mutations in the human Jagged1 gene are responsible for Alagille syndrome. Nat Genet. 1997;16:235–42. 9207787 10.1038/ng0797-235

[5] McDaniell R, Warthen DM, Sanchez-Lara PA, et al. NOTCH2 mutations cause Alagille syndrome, a heterogeneous disorder of the notch signaling pathway. Am J Hum Genet. 2006;79:169–73. 16773578 10.1086/505332 PMC1474136

[6] Vandriel SM, Li LT, She H, et al. Natural history of liver disease in a large international cohort of children with Alagille syndrome: results from the GALA study. Hepatology. 2023;77:512–29. 36036223 10.1002/hep.32761 PMC9869940

[7] Ellegaard O, Wallin JA. The bibliometric analysis of scholarly production: How great is the impact? Scientometrics. 2015;105:1809–31. 26594073 10.1007/s11192-015-1645-z PMC4643120

[8] Li Y, Li L, Yin W, Wan J, Zhong X. Bibliometric analysis of the correlation between H. pylori and inflammatory bowel disease. JGH Open. 2024;8:e70014. 39148512 10.1002/jgh3.70014 PMC11325047

[9] Akmal M, Hasnain N, Rehan A, et al. Glioblastome multiforme: a bibliometric analysis. World Neurosurg. 2020;136:270–82. 31953095 10.1016/j.wneu.2020.01.027

[10] Sokol RJ, Heubi JE, Balistreri WF. Intrahepatic “cholestasis facies”: Is it specific for Alagille syndrome? J Pediatr. 1983;103:205–8. 6875709 10.1016/s0022-3476(83)80345-x

[11] Masek J, Andersson ER. Jagged-mediated development and disease: mechanistic insights and therapeutic implications for Alagille syndrome. Curr Opin Cell Biol. 2024;86:102302. 38194749 10.1016/j.ceb.2023.102302

[12] Gilbert MA, Bauer RC, Rajagopalan R, et al. Alagille syndrome mutation update: comprehensive overview of JAG1 and NOTCH2 mutation frequencies and insight into missense variant classification. Hum Mutat. 2019;40:2197–220. 31343788 10.1002/humu.23879 PMC6899717

[13] Karpen SJ, Kamath BM, Alexander JJ, et al. Use of a comprehensive 66-gene cholestasis sequencing panel in 2171 cholestatic infants, children, and young adults. J Pediatr Gastroenterol Nutr. 2021;72:654–60. 33720099 10.1097/MPG.0000000000003094

[14] Ayoub MD, Bakhsh AA, Vandriel SM, Keitel V, Kamath BM. Management of adults with Alagille syndrome. Hepatol Int. 2023;17:1098–112. 37584849 10.1007/s12072-023-10578-x PMC10522532

[15] Gala Study Grp, Vandriel S, Li L, et al. Phenotypic divergence of JAGGED1 and NOTCH2-associated alagille syndrome: results from the international multicenter GALA study group. Hepatology. 2020;72:882A-A884.

[16] Adams JM, Huppert KA, Castro EC, et al. Sox9 is a modifier of the liver disease severity in a mouse model of Alagille syndrome. Hepatology. 2020;71:1331–49. 31469182 10.1002/hep.30912 PMC7048647

[17] Zhao C, Lancman JJ, Yang Y, et al. Intrahepatic cholangiocyte regeneration from an Fgf-dependent extrahepatic progenitor niche in a zebrafish model of Alagille syndrome. Hepatology. 2022;75:567–83. 34569629 10.1002/hep.32173 PMC8844142

[18] Lykavieris P, Hadchouel M, Chardot C, Bernard O. Outcome of liver disease in children with Alagille syndrome: a study of 163 patients. Gut. 2001;49:431–5. 11511567 10.1136/gut.49.3.431 PMC1728437

[19] Li J, Wu H, Chen S, et al. Clinical and genetic characteristics of Alagille syndrome in adults. J Clin Transl Hepatol. 2023;11:156–62. 36406308 10.14218/JCTH.2021.00313 PMC9647109

[20] Schindler EA, Gilbert MA, Piccoli DA, Spinner NB, Krantz ID, Loomes KM. Alagille syndrome and risk for hepatocellular carcinoma: need for increased surveillance in adults with mild liver phenotypes. Am J Med Genet A. 2021;185:719–31. 33369123 10.1002/ajmg.a.62028 PMC7898517

[21] Send SR. Nutritional management of cholestasis. Clin Liver Dis (Hoboken). 2020;15:9–12. 32104570 10.1002/cld.865 PMC7041952

[22] Karpen SJ, Kelly D, Mack C, Stein P. Ileal bile acid transporter inhibition as an anticholestatic therapeutic target in biliary atresia and other cholestatic disorders. Hepatol Int. 2020;14:677–89. 32653991 10.1007/s12072-020-10070-w

[23] Himes R, Rosenthal P, Dilwali N, Smith K, Venick R, Gonzalez-Peralta RP. Real-world experience of maralixibat in Alagille syndrome: novel findings outside of clinical trials. J Pediatr Gastroenterol Nutr. 2024;78:506–13. 38334237 10.1002/jpn3.12101

[24] Wang KS, Tiao G, Bass LM, et al. Analysis of surgical interruption of the enterohepatic circulation as a treatment for pediatric cholestasis. Hepatology. 2017;65:1645–54. 28027587 10.1002/hep.29019 PMC5397365

[25] Arnon R, Annunziato R, Schiano T, et al. Orthotopic liver transplantation for adults with Alagille syndrome. Clin Transplant. 2012;26:E94–100. 22211770 10.1111/j.1399-0012.2011.01574.x

[26] Menon J, Shanmugam N, Vij M, Rammohan A, Rela M. Multidisciplinary management of Alagille syndrome. J Multidiscip Healthc. 2022;15:353–64. 35237041 10.2147/JMDH.S295441 PMC8883402

